# Identification of differential gene expression profile from peripheral blood cells of military pilots with hypertension by RNA sequencing analysis

**DOI:** 10.1186/s12920-018-0378-2

**Published:** 2018-07-11

**Authors:** Xing-Cheng Zhao, Shao-Hua Yang, Yi-Quan Yan, Xin Zhang, Lin Zhang, Bo Jiao, Shuai Jiang, Zhi-Bin Yu

**Affiliations:** 1 Department of Aerospace Physiology, Fourth Military Medical University, Changle West Road 169#, Xi’an, 710032 People’s Republic of China; 2 Lintong Aviation Medical Evaluating and Training Center of Air Force, Xi’an, 710600 China; 3 Department of Traditional Chinese Medicine, Xijing Hospital, Fourth Military Medical University, Xi’an, 710032 China

**Keywords:** Hypertension, Military pilots, Peripheral blood cells, RNA sequencing

## Abstract

**Background:**

Elevated blood pressure is an important risk factor for cardiovascular disease and is also an important factor in global mortality. Military pilots are at high risk of cardiovascular disease because they undergo persistent noise, high mental tension, high altitude hypoxia, high acceleration and high calorie diet. Hypertension is the leading cause of cardiovascular disease in military pilots. In this study, we want to identify key genes from peripheral blood cells of military pilots with hypertension. Identification of these genes may help diagnose and control hypertension and extend flight career for military pilots.

**Methods:**

We use RNA sequencing technology, bioinformatics analysis and Western blotting to identify key genes from peripheral blood cells of military pilots with hypertension.

**Results:**

Our study detected 121 up-regulated genes and 623 down-regulated genes in the peripheral blood mononuclear cells (PBMCs) from hypertensive military pilots. We have also identified 8 important genes (*NME4*, *PNPLA7*, *GGT5*, *PTGS2*, *IGF1R*, *NT5C2*, *ENTPD1* and *PTEN*), a number of gene ontology categories and biological pathways that may be associated with military pilot hypertension.

**Conclusions:**

Our study may provide effective means for the prevention, diagnosis and treatment of hypertension for military pilot and extend their flight career.

**Electronic supplementary material:**

The online version of this article (10.1186/s12920-018-0378-2) contains supplementary material, which is available to authorized users.

## Background

Elevated blood pressure (BP) is an important risk factor of cardiovascular disease (CVD) and is also an important factor in global mortality, leading to about 9.4 million deaths per year [1]. The morbidity and mortality of CVD are associated with degrees of increased BP. For every increase of 20 mmHg in the systolic BP above 115 mmHg, the incidence of CVD risk will double [2]. In general, the threshold of hypertension is set to systolic pressure of 140 mmHg (150 mmHg for older adults) or diastolic pressure of 90 mmHg based on BP measurement in quiescent condition [3]. One-third American adults over 18 years old of age have hypertension, and 54% of old people (55- to 64-year-olds) have high BP. Among those over 75 years old of age in the United States, nearly 80% have hypertension [4].

Military pilots are at high risk of CVD because they are undergo persistent noise, high mental tension, high altitude hypoxia, high acceleration and high calorie diet [5]. Hypertension is the leading cause of CVD in military pilots. Although hypertension itself will not cause sudden disability in flight, but it is a main risk factor for disability in flight career and it is also one of the major reasons to cause the pilot grounded [6]. Wenzel et al. reported that the incidence of hypertension in Brazilian Air Force is about 22% [7]. Grossman et al. found 2.4% of the pilots had moderate or higher blood pressure in a 7.5-year follow-up study of Israeli Air Force pilots [8]. The hypertension prevalence rate was 9.7% in Chinese Air Force pilots, and the grounded rate of pilots was 21.7% among students in the flight academy because of hypertension or increased blood pressure [9]. Essential hypertension is a disease caused by complex, multifactorial and multigenic changes, and it is the result of both gene regulation and environmental impact [10]. In this study, we use RNA sequencing technology, bioinformatics analysis and Western blotting to identify key genes from peripheral blood cells of military pilots with hypertension. Identification of these genes may help diagnose and control hypertension and extend flight career for military pilots.

## Methods

### Study subject

For RNA-Seq, six samples of peripheral blood cell from military pilot (3 hypertensives and 3 normotensives) were collected. For quantitative RT-PCR and Western blotting analysis, another 8 samples of peripheral blood cell from fighter pilot (4 hypertensives and 4 normotensives) were collected. All samples collected with the help of doctors from Lintong Aviation Medical Evaluating and Training Center of Air Force, Xi’an, China. The average systolic blood pressure (SBP) of these hypertension pilots was above 160 mmHg and the average diastolic blood pressure (DBP) was above 100 mmHg. The average SBP of these normotensive pilots was below 135 mmHg and the average DBP was below 85 mmHg.

### Peripheral blood mononuclear cells (PBMCs) isolation

Five milliliter whole blood collected was transferred to a 15 ml sterile centrifuge tube, 5 ml phosphate buffer saline (PBS) was added to dilute the whole blood. Five milliliter lymphocyte separation medium was added to another 15 ml sterile centrifuge tube. The diluted whole blood was transferred to the lymphocyte separation medium gently to avoid mixing. Then the blood was centrifuged at 2000 rpm for 20 min, room temperature. The white membrane cells of PBMSs were sucked into another sterile 15 ml centrifuge tube. Add 5 ml PBS and centrifuge at 1000 rpm for 10 min. Aspirate supernatant and add 2 ml ACK buffer to the tube, suspend the cells and keep standing for 5 min. Add 5 ml PBS and centrifuge at 1000 rpm for 10 min. Aspirate the supernatant, and the cells were quickly frozen in liquid nitrogen and used for further analysis.

### RNA sequencing

RNA sequencing was entrusted to Novel Bioinformatics Co., Ltd., Shanghai, China. Using high-throughput Life technologies Ion Proton Sequencer, the transcript with poly(A)-containing RNA of Human were analyzed.

### Quality control

Fast - QC software (http://www.bioinformatics.babraham.ac.uk/projects/fastqc/) was employed to evaluate the overall quality of sequencing data.

### Gene expression calculation

The expression of genes is mainly calculated by RPKM (Reads Per Kb per Million reads) method. The formula is: $RPKM=\frac{10^6C}{NL/{10}^3}$ RPKM is gene expression, C is the unique number of reads on the gene, and N is the unique number of total reads in the reference gene, and L is the base number of the gene coding region. RPKM method can eliminate the effects of gene length and sequencing on the expression of genes, which can be directly used to compare the gene expression differences between different samples.

### Principal component analysis

We applied the PCA analysis based the whole gene expression table and the R script utilizing following package: MASS, evd, rgl and pvclust. Command we used was described as followings:

R script:

dat < − read.table(“all.rpkm.exp.txt”,sep = “\t”, header = T).

colnameall = colnames(dat).

colname = colnames(dat[,2:length(colnameall)]).

dat.pca < −princomp(dat[2:length(colnameall)]).

summary(dat.pca).

plot<−plot3d(dat.pca$loadings[,1],dat.pca$loadings[,2],dat.pca$loadings[,3],type = “s”,col. = col.,size = 0.8,xlab = “PC1”,ylab = “PC2”,zlab = “PC3”).

texts3d(dat.pca$loadings[,1],dat.pca$loadings[,2]-0.02,dat.pca$loadings[,3],texts = colname,font = 5).

### GO analysis

The difference of gene analysis based on the database from BP, MF, CC GO annotation in the three dimensions and all GOs were obtained. Each GO significance level was obtained by using the Fisher test (*P* Value) and so gene enrichment significant difference GOs were screened out [11, 12].

### Pathway analysis

The differential expression genes filter out were annotated in KEGG database (http://www.genome.jp/kegg/) for Pathway annotations, and all the Pathway Terms of different genes involved were got. The Pathway of significance level is obtained by using the Fisher test (P Value), and the significant Pathway Term of differential expression gene enrichment was screened out.

### Gene-act-network

The construction of gene interaction is to sort out the regulation of all the genes, and through the construction of signal transduction network, we could easily find the vein of gene signal transduction. Based on KEGG database, we could get gene interactions. So, we constructed gene and adjacency matrix using gene interactions [13–17].

### Western blotting

PBMCs were isolated as before. Total protein was extracted by using the RIPA reagent (Beyotime, Shanghai, China). The sample protein concentration was tested by measured by BCA Protein Assay reagents (Thermo Scientific, Rockford, IL). After the protein electrophoresis, the samples were transferred to the PVDF membrane, which was then incubated for primary antibodies overnight at 4 °C, and then the membrane was incubated for horseradish peroxidase (HRP)-conjugated secondary antibodies for 2 h at room temperature. The primary antibodies used were as follows: *NT5C2* rabbit polyclonal antibody (Proteintech, Wuhan, China), *ENTPD1* rabbit polyclonal antibody (Proteintech, Wuhan, China), GGT5 rabbit polyclonal antibody (Proteintech, Wuhan, China), *COX2* (*PTGS2*) rabbit polyclonal antibody (Proteintech, Wuhan, China), *PTEN* rabbit polyclonal antibody (Proteintech, Wuhan, China), *IGF1R* rabbit polyclonal antibody (Proteintech, Wuhan, China), rabbit anti-*NME4* (Bioss Antibodies, Beijing, China), rabbit anti-*PLCG2* (Bioss Antibodies, Beijing, China), rabbit anti-PI3 Kinase p110 delta (*PIK3D*) (Bioss Antibodies, Beijing, China), anti-*PNPLA7* (Santa Cruz Biotechnology, Dallas, TX), anti-*β-actin* (Santa Cruz Biotechnology, Dallas, TX). The membrane was chemiluminescence using the chemiluminescent reagents (Millipore Corporation, Billerica, MA) and image-forming system Tanon 4200 (Tanon Science & Technology Co., Ltd., Shanghai, China).

## Results

### Overview of RNA sequencing data

**Fig. 1:**
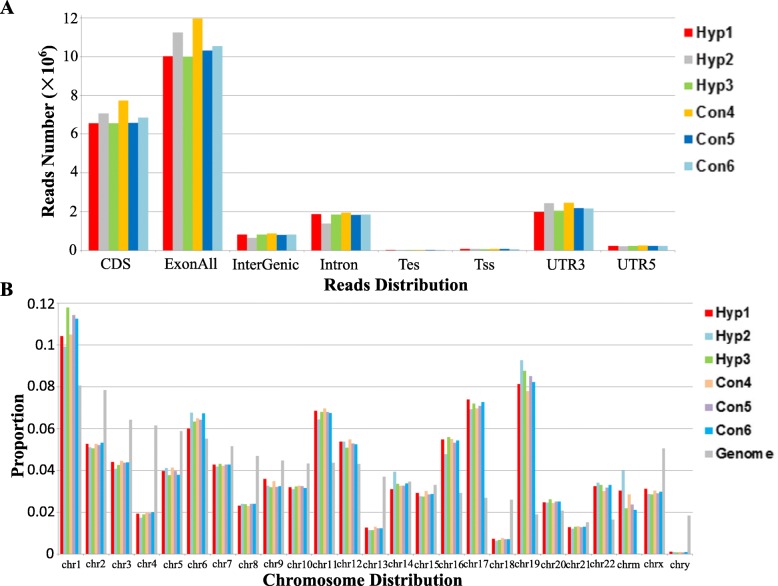
An overview of Reads Distribution and Chromosome Distribution of RNA sequencing. **a** Reads number onto the regions of CDS, exons, intergenic region (InterGenic), introns, transcription end site (TES), transcription start site (TSS), 3’-UTR and 5’-UTR. **b** Distribution of reads on chromosomes

We used Fast-QC online software (http://www.bioinformatics.babraham.ac.uk/projects/fastqc/) to assess the quality of sequencing results (Results were not shown). The results showed that the sequencing data of all samples were qualified. Total raw reads of these six samples were about 12–15 million. GC content of these six samples was about 53%. The average reads mapped to human genome sequence were about 1.28 ± 0.78 × 10^7^reads (96% of the total reads) in the six samples (Additional file 1: Table S1) and about 92% of the total reads (1.24 ± 0.74 × 10^7^ reads) were mapped to human genome sequence uniquely. MapSplice was employed to map the reads. According to the mapping results, about 1 × 10^7^ reads were mapped to the transcript exon, 7 × 10^6^ reads were mapped to CDS, 2 × 10^6^ reads were mapped to intron, 2 × 10^6^ reads were mapped to the UTR regions and the rest reads (about 5% or less) were mapped to intergenic regions, TSS (transcription start site) and TES (transcription end site) (Fig. 1a). We also detected the distribution on chromosomes of these sample mapped reads (Fig. 1b). The results showed that the most reads were aligned to chromosome 1 (about 10% or more) and the least reads were aligned to chromosome Y (less than 0.2%).

### Differential gene expression profiles between PBMCs from hypertensives and normotensives military pilots and GO analysis

The six samples were screened for difference gene expression with 3:3. The total number was 26, lower than the minimum standard of data analysis. Two discrete samples (Con4 and Hyp3) were deleted according to results of principal component analysis, and 744 differential genes were acquired. This number was enough for further analysis, so we adopted difference gene expression screened with 2:2.

**Fig. 2:**
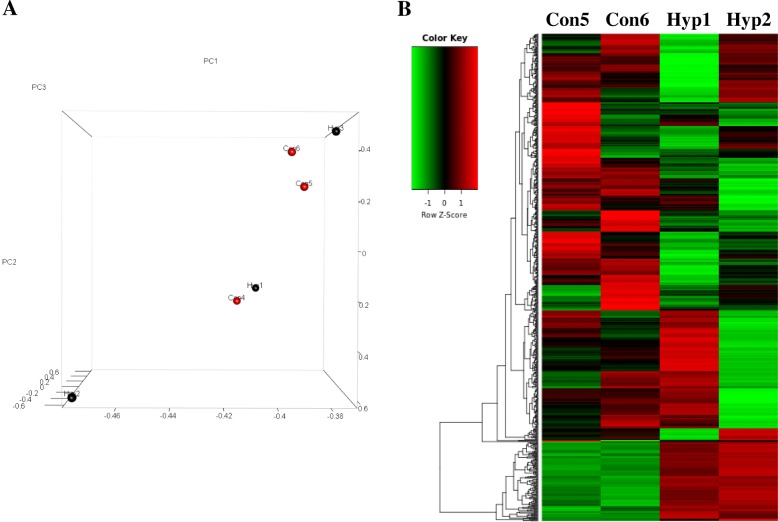
Clustering of differentially expressed genes. **a** PCA cluster analysis of these 6 samples. **b** Gene thermal map of differentially expressed genes

**Fig. 3:**
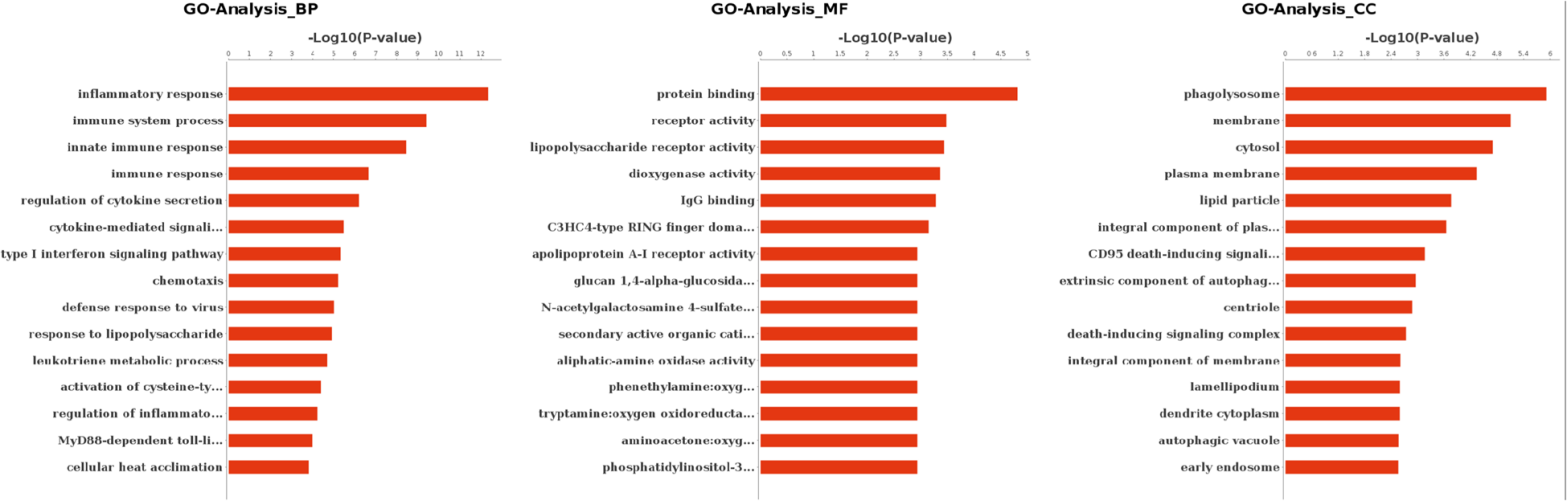
Gene Ontology (GO) Analysis of differentially expressed genes. GO analysis was annotated from three levels: BP, MF and CC. -Log_10_(*P*-value) was showed at abscissa axis and GO terms was showed at longitudinal axis. *P* value < 0.05 for all significant GO terms. BP: Biological Process; CC: Cellular Component; MF: Molecular Function

To characterize the gene expression changes between hypertensives and normotensives military pilots’ PBMCs, differentially expressed genes were screened by the following standard: Log_2_FC > 0.585 or Log_2_FC < − 0.585, FDR < 0.05 and *P* value < 0.05. We found 744 differentially expressed genes between hypertensives and normotensives military pilots’ PBMCs. Of these genes, 121 genes were up-regulated in hypertensives military pilots’ PBMCs and 623 genes were down-regulated. PCA (principal components analysis) cluster analysis was used to compare differential expression of these two groups. The differential gene expression patterns of these samples were showed by gene thermal map (Fig. 2). Gene ontology analysis was used to seek the functions of these differentially expressed genes. The results showed that there were 337 genes belonged to protein binding and 64 genes belonged to ATP binding for molecular function (MF). There were 91 genes belonged to signal transduction and 70 genes belonged to small molecule metabolic process for biological process (BP). As for cellular component (CC), there were 311 genes belonged to membrane and 248 genes belonged to cytoplasm. Results from GO term analyzing showed that inflammatory response, protein binding and phagolysosome were the most significant for BP, MF and CC respectively (Fig. 3).

### Pathways analysis of differential expression genes

**Fig. 4:**
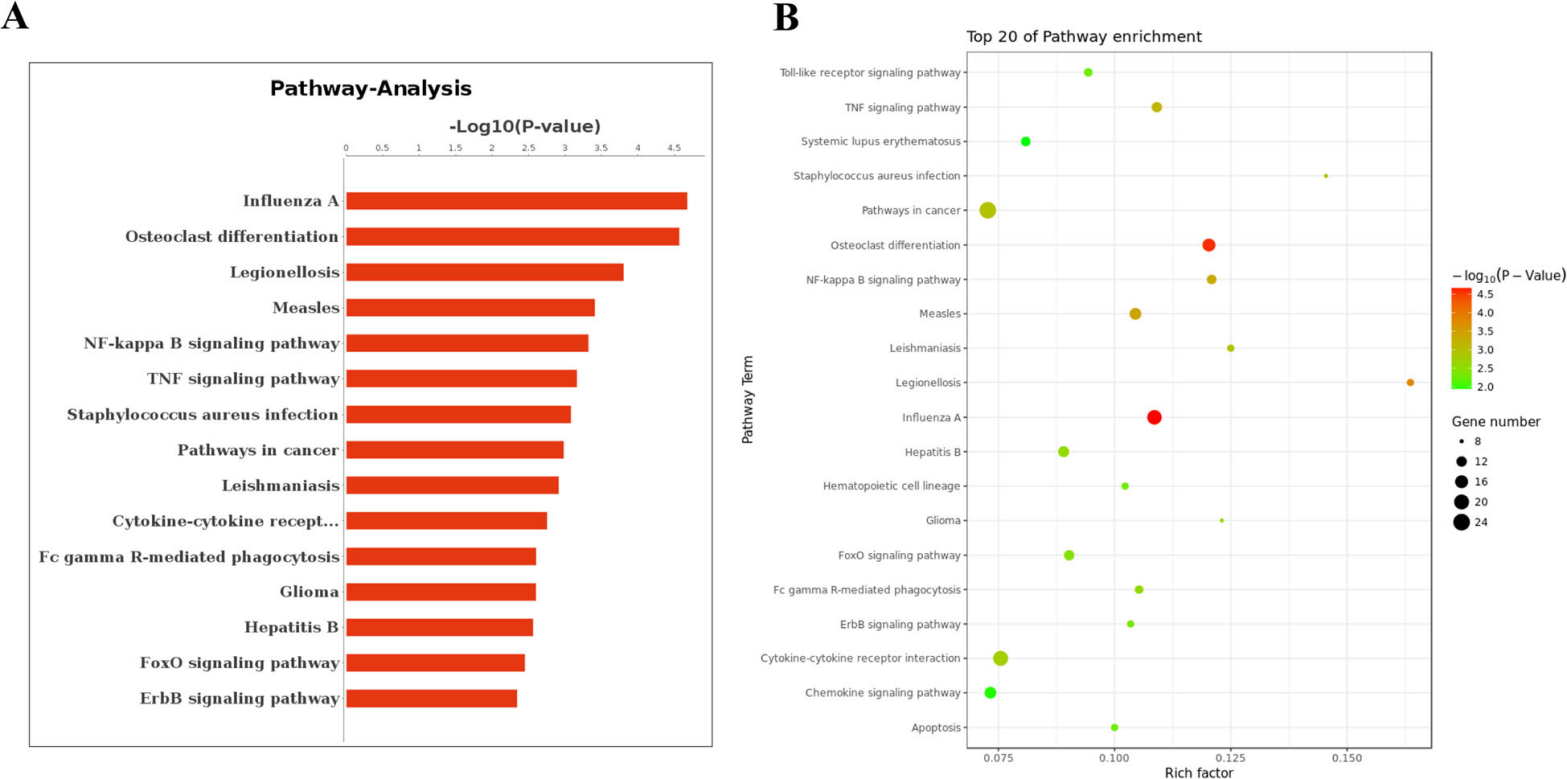
Pathway enrichment analysis of differentially expressed genes based on KEGG. **a** Histogram of pathway enrichment. -Log_10_(P-value) was showed at abscissa axis and pathway terms of KEGG was showed at longitudinal axis. **b** Top 20 of pathway enrichment was displayed by different colors and different sizes of the circle

The differential expression genes were involved in multiple GO, so we constructed functional relation network with significant GO-Term (*p*-value< 0.05) to reveal relationship between genes clearly based on hierarchical structure of GO (Additional file 2: Figure S1). Of these pathways, protein phosphorylation, toll-like receptor signaling pathway and cell surface receptor signaling pathway were in the core position (Additional file 2: Figure S1). Then, pathway-analysis was carried out to detect significant and important pathways of these differential expression genes. Influenza A and osteoclast differentiation were the most significant (Fig. 4a). Also, the top 20 of pathway enrichment was displayed in Fig. 4b. *P*-Value and gene number was indicated as circle size and color. Next, the pathways interaction network was built to analysis deeply. The analysis results showed that the most important pathways were apoptosis, Jak-STAT signaling pathway, toll-like receptor signaling and cytokine-cytokine receptor interaction (Additional file 3: Figure S2). Because these four pathways are located at the center of the all significant pathways and have the most arrowheads around, these four pathways are likely to be most important in the elevated blood pressure of military pilots. This result suggested that differential expression genes related to apoptosis, Jak-STAT signaling pathway, toll-like receptor signaling and cytokine-cytokine receptor interaction may have important role in the occurrence and development of elevated blood pressure of military pilots.

### Gene act network of differentially expressed genes

**Fig. 5:**
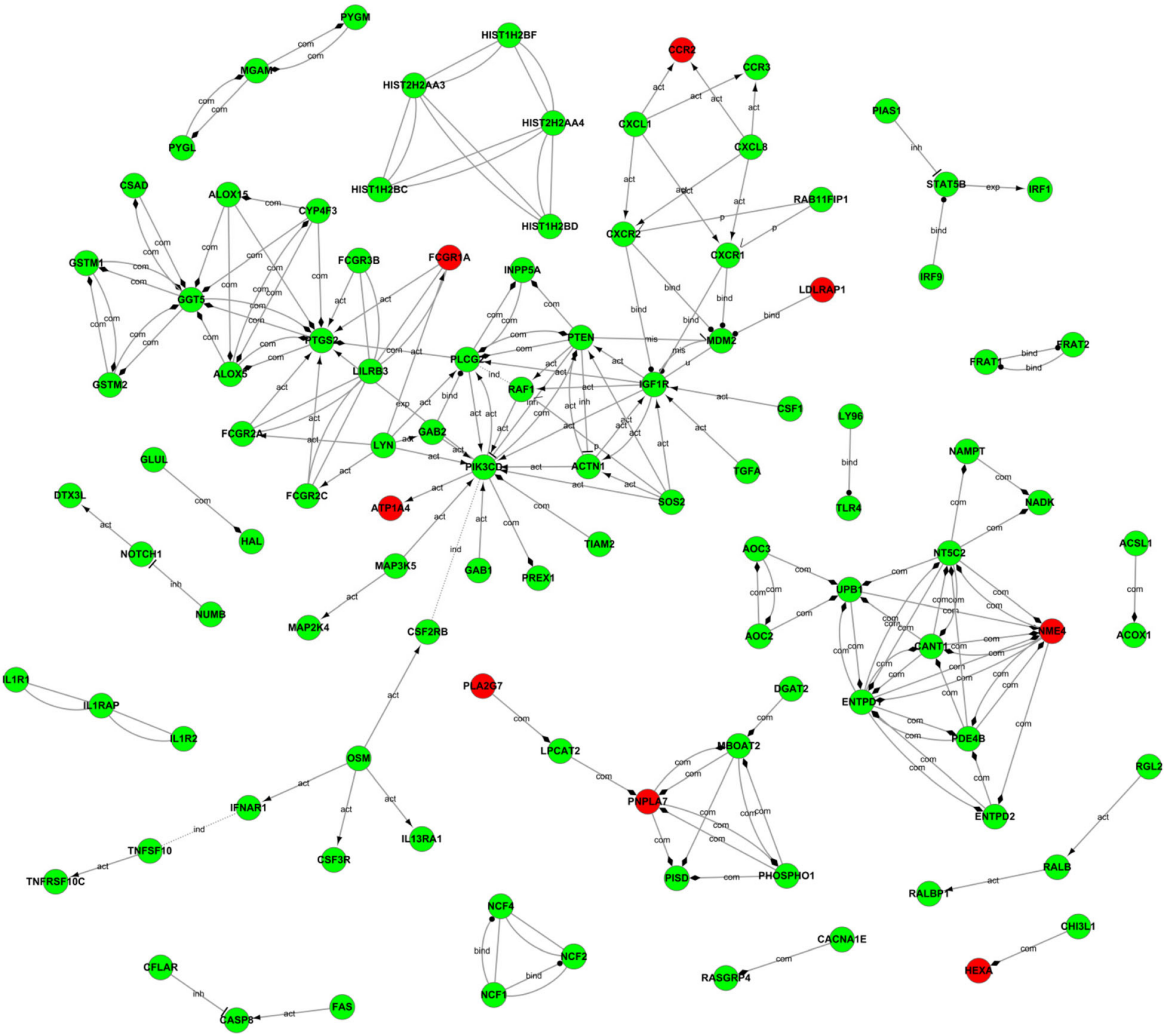
Gene Act network analysis. Red circles represent up-regulated genes in hypertension group; Green circles represent down-regulated genes in hypertension group, arrows indicate the direction of regulation

Although we got four important pathways related to elevated blood pressure of military pilots, we know that one gene may be involved in multiple signal transduction pathways at the same time. So next, we built gene act network based on the relationships between the differentially expressed genes including expression, binding, inhibition, activation and compound. This analysis method can form the corresponding regulation relationship between gene and gene and is easier to find important related genes under the intervention measures. By analysis the gene act network, we found that *NME4*, *PNPLA7*, *GGT5*, *PTGS2*, *IGF1R*, *PLCG2*, *NT5C2*, *ENTPD1*, *PIK3CD* and *PTEN* these ten genes were located at the center of the all significant genes and have the most arrowheads around (Fig. 5). And also, these ten genes were involved in apoptosis, Jak-STAT signaling pathway, toll-like receptor signaling and cytokine-cytokine receptor interaction signaling pathway previously mentioned. So Next, we will confirm changes of these genes in military pilots’ PBMCs of hypertensives and normotensives with Western blotting.

### Validation of representative differentially expressed genes by western blotting

**Fig. 6:**
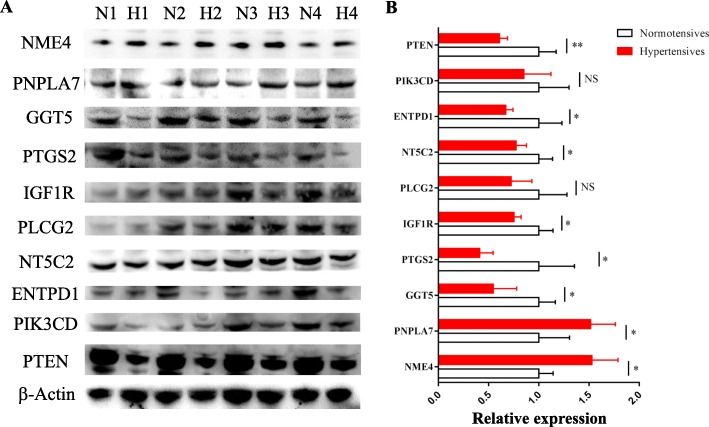
Western blotting validation of relative expression levels of representative differentially expressed genes. **a** Western blotting of 10 representative differentially expressed genes from 4 hypertensive (H1, H2 H3 and H4) and 4 normotensive (N1, N2, N3, N4) military pilots. **b** Statistics of relative expression of Western blotting. Bars = means ± SD. ^*^P < 0.05, ^**^P < 0.01, NS, not significant, *n* = 4

The expression of *NME4*, *PNPLA7*, *GGT5*, *PTGS2*, *IGF1R*, *PLCG2*, *NT5C2*, *ENTPD1*, *PIK3CD* and *PTEN* from PBMCs of hypertensive and normotensive military pilots were detected by Western blotting. The results from Western blotting showed that NME4 and PNPLA7 these two genes were up-regulated significantly and *GGT5*, *PTGS2*, *IGF1R*, *NT5C2*, *ENTPD1* and *PTEN* these six genes were down-regulated significantly in PBMCs of military pilots with hypertension (Fig. 6). Although the expression change of *PLCG2* and *PIK3CD* was not significant between hypertensives and normotensives, there was downward trend in PBMCs of hypertensive military pilots (Fig. 6). The results indicated that the expression change of *NME4*, *PNPLA7*, *GGT5*, *PTGS2*, *IGF1R*, *NT5C2*, *ENTPD1* and *PTEN* could be as sign of elevation of blood pressure for military pilots.

## Discussion

Military pilots are in a state of persistent noise, high mental tension, high altitude hypoxia, high acceleration and their high calorie diet, so hypertension is a very common disease in this group. To identify the key genes that related to hypertension of military pilots is very necessary for prevention, diagnosis and treatment of this disorder. In this study, we used RNA sequencing of PBMCs to identify differential gene expression profile between the hypertensive and normotensive military pilots.

Six samples were sequenced and the difference gene expression was compared with 3:3. Because two samples (Con4 and Hyp3) were discrete, so we took them out and adopted different gene expression screened with 2:2. 121 up-regulated genes and 623 down-regulated genes were identified as different expressed genes between hypertensive and normotensive military pilots. We selected 10 important and significant genes according to results of gene act network analysis. Western blotting was employed to validate the expression change of the 10 genes above. The results showed that expression change of *NME4*, *PNPLA7*, *GGT5*, *PTGS2*, *IGF1R*, *NT5C2*, *ENTPD1* and *PTEN* were consistent with the results of RNA sequencing.

*NME4* (also known as *NDPK-D*, *Nm23-H4*) belongs to the *Nm23* family, which includes 10 isoforms (*NME1* to *NME10*) [18]. The classical function of *NME4* as a group I isoform is its NDP kinase activity [19]. *PNPLA7* (also known as *NTE-R1* or *NRE*) is a member of the *PNPLAs* family, and its encoded protein is very conservative in mice, rats and human. *PNPLA7* plays an important role in the hydrolysis of triglycerides, energy metabolism, lipid formation and adipocyte differentiation [20]. Up-regulation of *PNPLA7* indicates elevated blood lipids, which has some correlation with hypertension.

The main expression of *Gamma-glutamyl transferase 5* (*GGT5*) is on the surface of cell membrane, and the role of *GGT5* is to hydrolyze the gamma-glutamyl bond glutathione [21]. There is no phenotypic abnormality in *GGT5* knockout mice under normal conditions. But *GGT5* gene knockout mice were unable to metabolize *LTC4*, resulting in diminished potential of neutrophils infiltrating into the peritoneum. The expression and function of *GGT5* in human are rarely reported [22]. *PTGS2*(also known as *COX-2*) could promote carcinogenesis and metastasis of multiple types of tumors. Expression of *PTGS2* could be dramatically up-regulated by high levels of noise exposure and high altitude hypoxia [23, 24]. Park et al. reported that high fat diet could reduce *PTGS2* expression [25], indicating that expression change of *PTGS2* may be caused by noise exposure, high altitude hypoxia and high fat diet. *IGFR* is a member of the receptor tyrosine family and can form homodimers with insulin receptor (InsR) to identify and bind to the ligand of insulin receptor *IGF1* and *IGF2* [26]. Heterozygous deficiency of *Igf1r* reduced postnatal growth and develop age-dependent insulin resistance. Old-aged *Igf1r*^*+/−*^ mice had increased adiposity and exhibited increased adipogenesis [27], indicated that reduced expression of *IGF1R* may have a correlation with hypertension. *NT5C2* plays an important role in purine metabolism. Some papers reported that there were some relationships between somatic mutations of *NT5C2* and T-acute lymphoblastic leukemias (T-ALL) [28, 29]. *ENTPD1* (also known as *CD39*) is a plasma membrane protein and its role is to hydrolyze extracellular ATP and ADP to AMP. Helenius et al. reported that the expression of *ENTPD1* was significantly reduced in small arterial endothelial cells in patients with pulmonary arterial hypertension (PAH). The attenuation function of *ENTPD1* is closely related to vascular dysfunction, suggested that *ENTPD1* may be a novel target for PAH therapy [30]. Their results strongly suggested that down-regulation of *ENTPD1* may be a sign of hypertension, consistent with our results. *PTEN* signaling pathway is one of the most important signaling pathways that regulate individual development, and participate in the process of cell proliferation, differentiation, aging and apoptosis. Schwerd et al. reported that mutation of *PTEN* led severe macrocephaly and mild intellectual disability in adolescent [31]. Skalska-Sadowska et al. reported that mutations of *PTEN* induced T-ALL [32]. These results suggested that abnormal expression of *PTEN* could lead nervous system abnormalities and hematologic disease, maybe have some relationship with hypertension.

Some of the above eight genes are associated with a high-fat diet, some are associated with noise and high-altitude hypoxia, some may be associated with high mental tension. We hypothesized that the expression change of these genes may affect the level of insulin-like growth factor and small vessel remodeling to induce hypertension under the long-term impact of flight environment. Therefore, we should pay attention to the expression changes of these eight genes in the prevention and control of military pilots’ hypertension. Early prevention and early treatment according to the expression change of these eight genes, may extend flight career of military pilots.

At last, we acknowledge that the lack of non-pilot controls is a limitation of the study. The eight differential genes we identified may not be pilots specific. Therefore, our results may lead to some errors in the prevention, diagnosis and treatment of pilot hypertension. But our study still has good guidance for the prevention, diagnosis, and treatment of hypertension in pilots, because the samples used in RNA sequencing and Western blotting experiments in this study are all from military pilots. We will employ non-pilot controls including hypertensives and normotensives to identify differential genes more accurate in the future.

## Conclusions

In summary, this study detected gene expression difference in PBMCs between military pilot hypertension and normotensives. We have also identified 8 important genes (*NME4*, *PNPLA7*, *GGT5*, *PTGS2*, *IGF1R*, *NT5C2*, *ENTPD1* and *PTEN*), a few GO categories and biological pathways that may be associated with the military pilot hypertension. Our study may provide effective means for the prevention, diagnosis and treatment hypertension of military pilot, extend their flight career.

## Additional files

Additional file 1: **Table S1.** Statistics of raw and mapped reads from RNA-seq analysis of PBMCs from hypertensives (Hyp) and normotensives (Con) military pilot. (PPTX 62 kb)

Additional file 2: **Figure S1.** Relationship between genes based on hierarchical structure of GO. (PPTX 664 kb)

Additional file 3: **Figure S2.** Pathways interaction network analysis. (PPTX 496 kb)

## Abbreviations

BP: Blood pressure
CC: Cellular component
CDS: Coding sequence
CVD: Cardiovascular disease
DBP: Diastolic blood pressure
FDR: False discovery rate
GO: Gene ontology
HRP: Horseradish peroxidase
MF: Molecular function
PBMCs: Peripheral Blood Mononuclear Cells
PBS: Phosphate buffer saline
PVDF: Polyvinylidene fluoride
RPKM: Reads per Kb per million reads
SBP: Systolic blood pressure
T-ALL: T-acute lymphoblastic leukemias
TES: Transcription end site
TSS: Transcription start site
