# Supplementary material for: Identification of differential gene expression profile from peripheral blood cells of military pilots with hypertension by RNA sequencing analysis
Source: BMC Med Genomics. 2018 Jul 11;11:59. doi: 10.1186/s12920-018-0378-2 (PMC6042441; doi:10.1186/s12920-018-0378-2)
Supplement: Supplementary file 1 — Table S1. Statistics of raw and mapped reads from RNA-seq analysis of PBMCs from hypertensives (Hyp) and normotensives (Con) military pilot. (PPTX 62 kb) [file 12920_2018_378_MOESM1_ESM.pptx]

## Slide 1
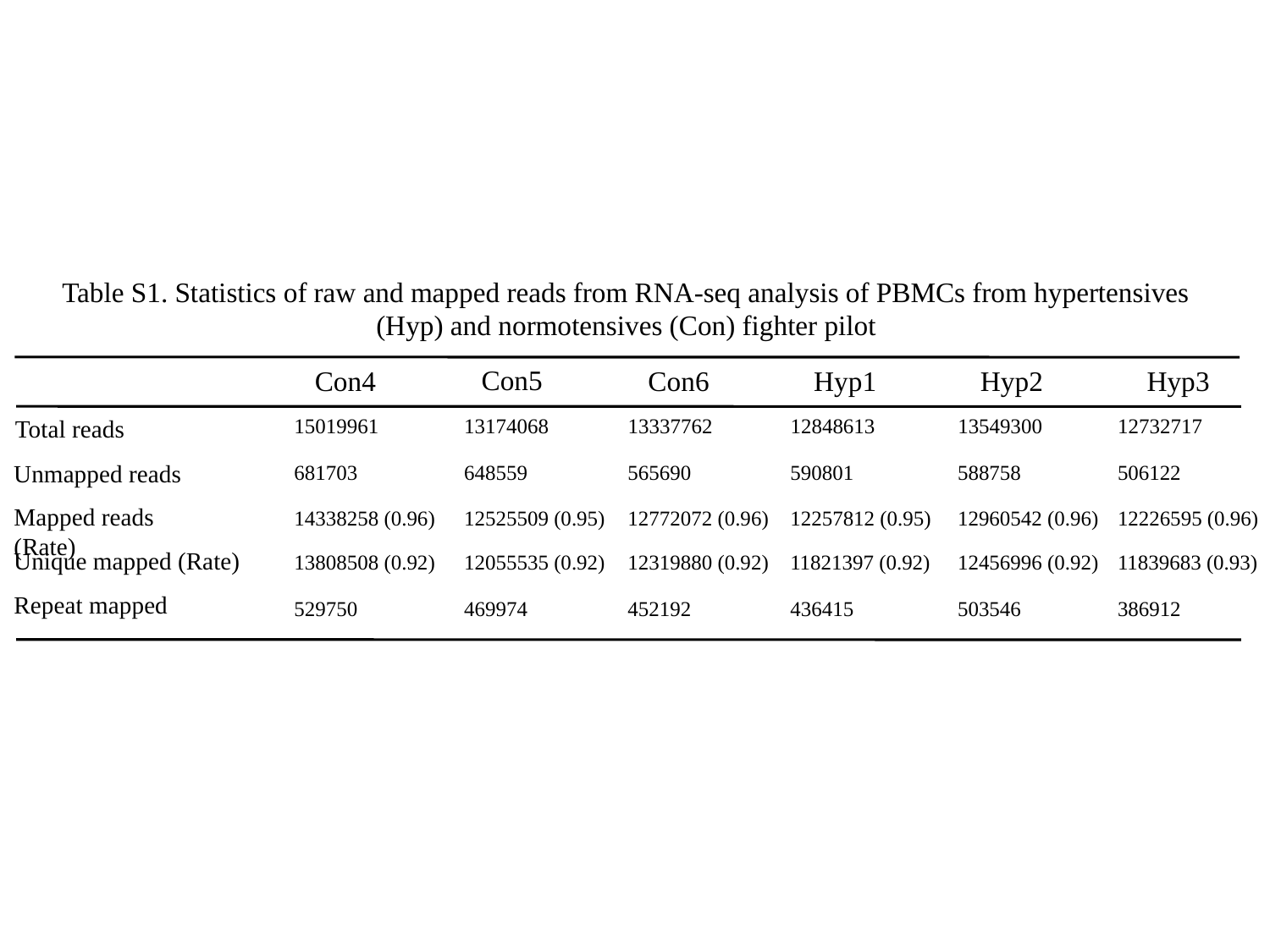

Table S1. Statistics of raw and mapped reads from RNA-seq analysis of PBMCs from hypertensives (Hyp) and normotensives (Con) fighter pilot
Con5
Hyp1
Con6
Hyp2
Hyp3
Con4
15019961
13174068
13337762
12848613
13549300
12732717
Total reads
Unmapped reads
681703
648559
565690
590801
588758
506122
Mapped reads (Rate)
14338258 (0.96)
12525509 (0.95)
12772072 (0.96)
12257812 (0.95)
12960542 (0.96)
12226595 (0.96)
Unique mapped (Rate)
13808508 (0.92)
12055535 (0.92)
12319880 (0.92)
11821397 (0.92)
12456996 (0.92)
11839683 (0.93)
Repeat mapped
529750
469974
452192
436415
503546
386912
