# Supplementary material for: Identification of differential gene expression profile from peripheral blood cells of military pilots with hypertension by RNA sequencing analysis
Source: BMC Med Genomics. 2018 Jul 11;11:59. doi: 10.1186/s12920-018-0378-2 (PMC6042441; doi:10.1186/s12920-018-0378-2)
Supplement: Supplementary file 2 — Figure S1. Relationship between genes based on hierarchical structure of GO. (PPTX 664 kb) [file 12920_2018_378_MOESM2_ESM.pptx]

## Slide 1
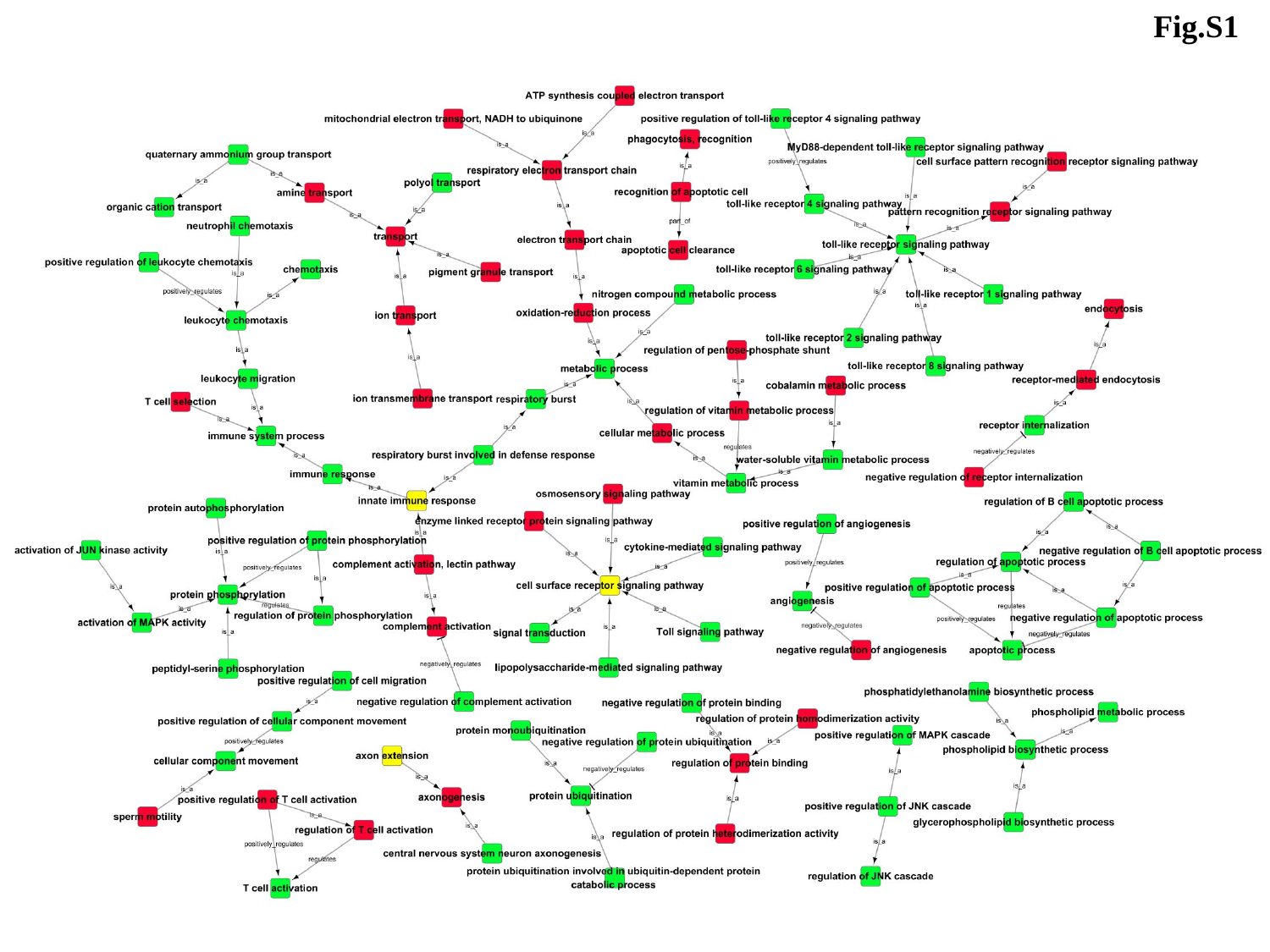

Fig.S1

## Slide 2
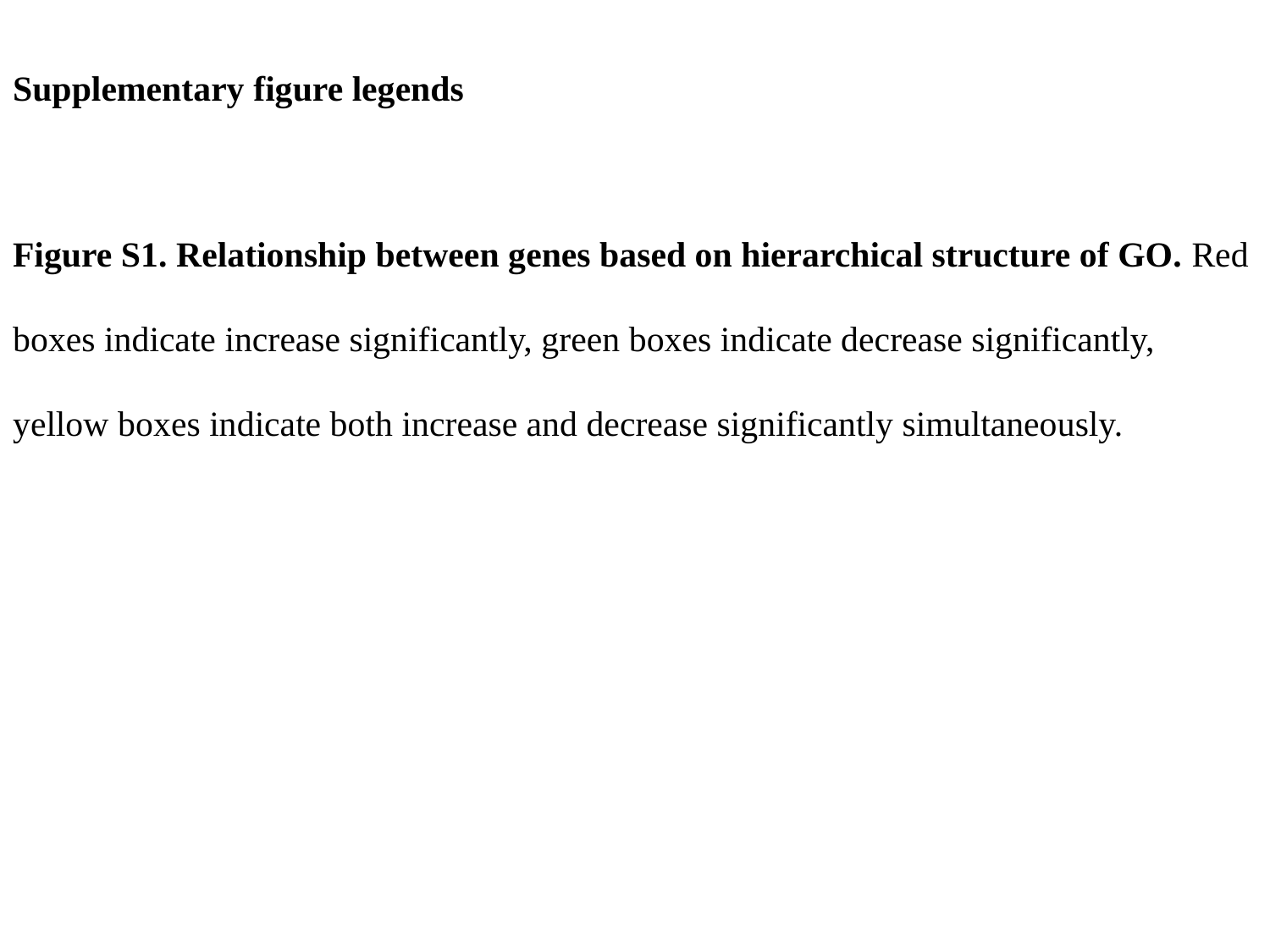

Supplementary figure legends
Figure S1. Relationship between genes based on hierarchical structure of GO. Red boxes indicate increase significantly, green boxes indicate decrease significantly, yellow boxes indicate both increase and decrease significantly simultaneously.
