# Supplementary material for: Identification of differential gene expression profile from peripheral blood cells of military pilots with hypertension by RNA sequencing analysis
Source: BMC Med Genomics. 2018 Jul 11;11:59. doi: 10.1186/s12920-018-0378-2 (PMC6042441; doi:10.1186/s12920-018-0378-2)
Supplement: Supplementary file 3 — Figure S2. Pathways interaction network analysis. (PPTX 496 kb) [file 12920_2018_378_MOESM3_ESM.pptx]

## Slide 1
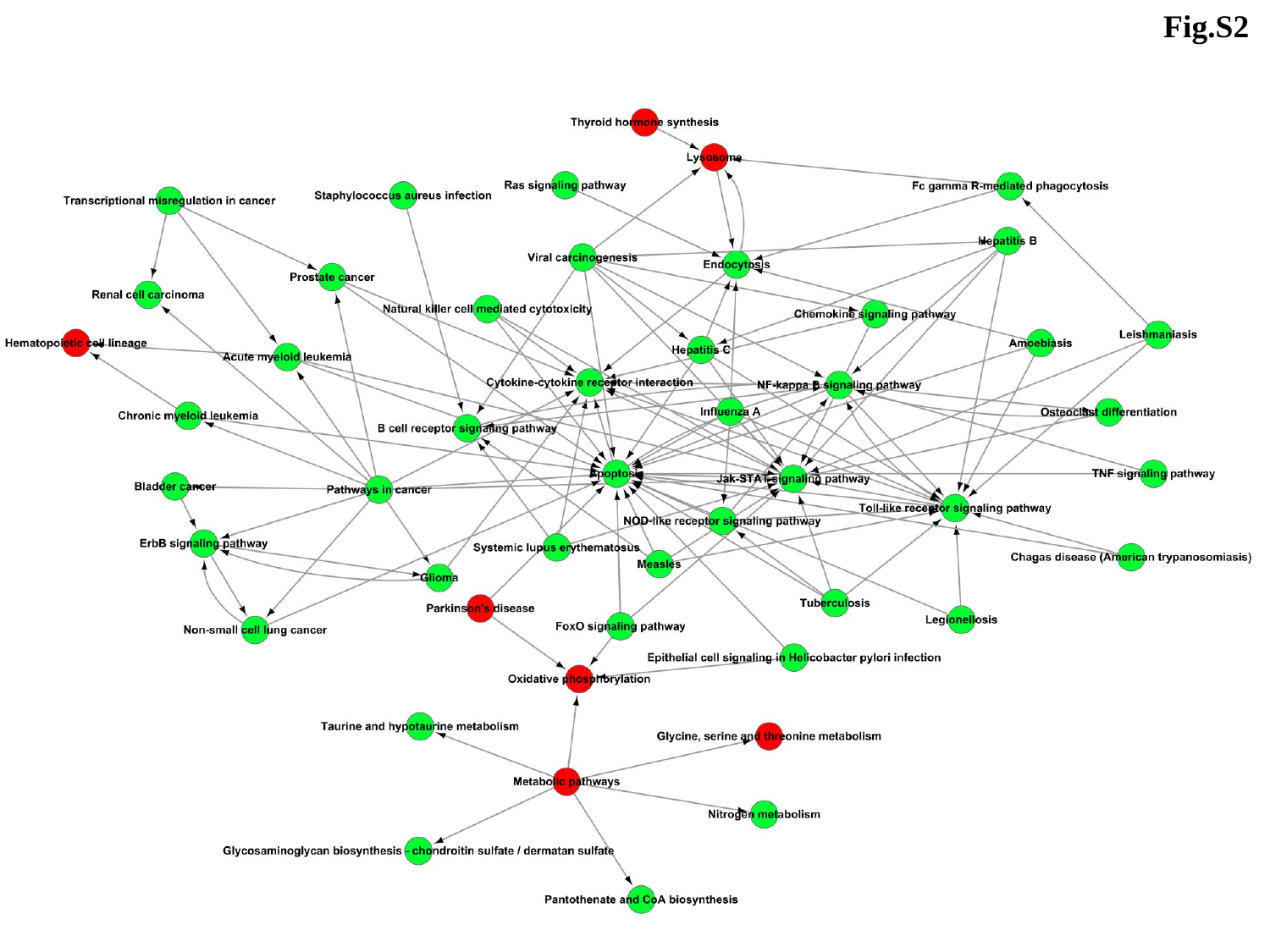

Fig.S2

## Slide 2
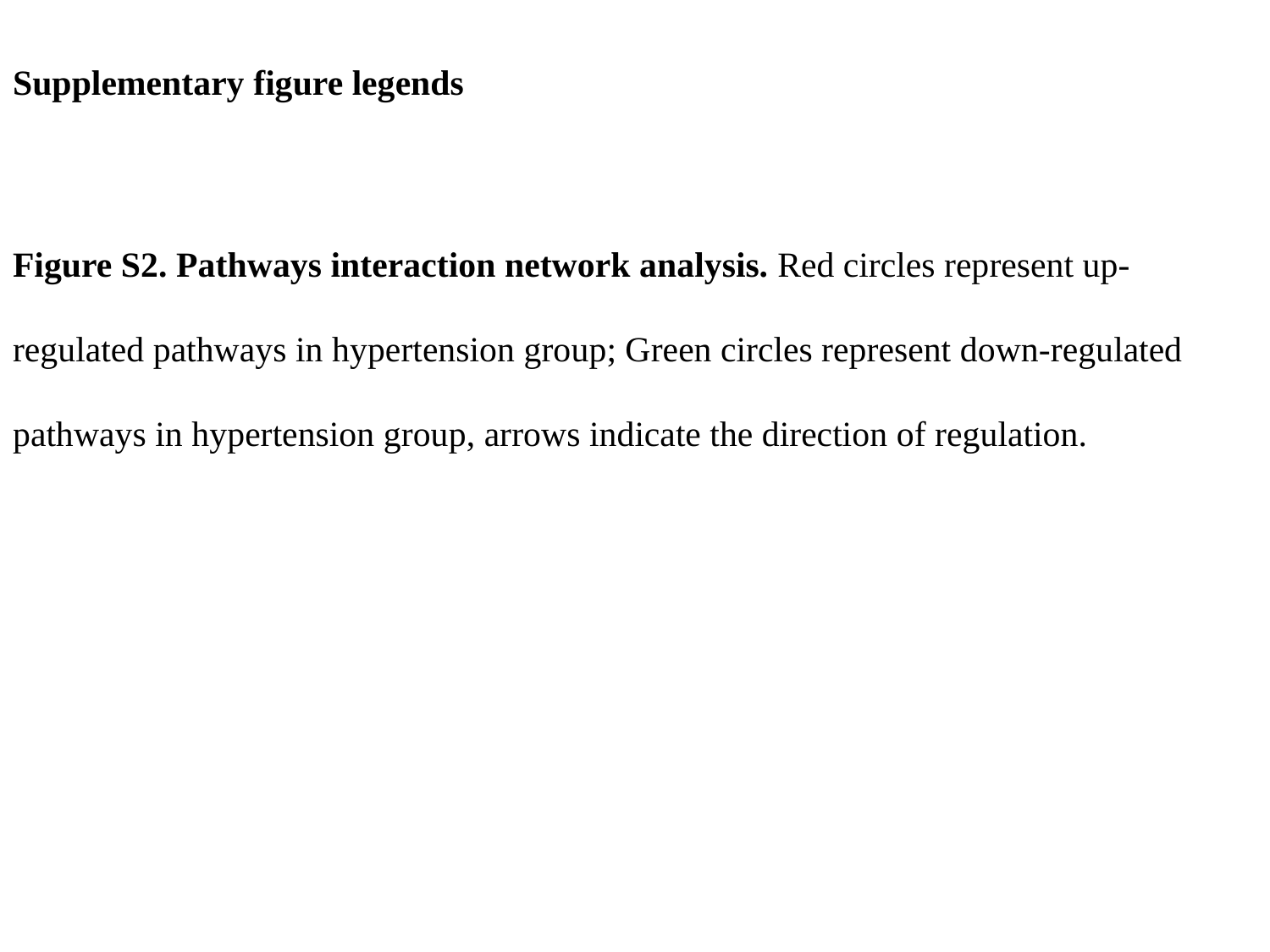

Supplementary figure legends
Figure S2. Pathways interaction network analysis. Red circles represent up-regulated pathways in hypertension group; Green circles represent down-regulated pathways in hypertension group, arrows indicate the direction of regulation.
